# Upcycling Fish By-Products into Bioactive Fish Oil: The Suitability of Microwave-Assisted Extraction

**DOI:** 10.3390/biom13010001

**Published:** 2022-12-20

**Authors:** José Pinela, Beatriz de la Fuente, Matilde Rodrigues, Tânia C. S. P. Pires, Filipa Mandim, André Almeida, Maria Inês Dias, Cristina Caleja, Lillian Barros

**Affiliations:** 1 Centro de Investigação de Montanha (CIMO), Instituto Politécnico de Bragança, Campus Santa Apolónia, 5300-253 Bragança, Portugal; 2 Laboratório Associado para a Sustentabilidade e Tecnologia em Regiões de Montanha (SusTEC), Instituto Politécnico de Bragança, Campus de Santa Apolónia, 5300-253 Bragança, Portugal; 3 Preventive Medicine and Public Health, Food Science, Toxicology and Forensic Medicine Department, Faculty of Pharmacy, Universitat de València, Avda, Vicent Andrés Estellés, 46100 València, Spain; 4 ITS—Indústria Transformadora de Subprodutos S.A., Rua Padre Adriano, 61, Santo Antão do Tojal, 2660-119 Loures, Portugal

**Keywords:** fatty acids, lipid quality indices, sonoextraction, Soxhlet extraction, antimicrobial activity, antioxidant activity, cytotoxic activity, process optimization, by-products valorization

## Abstract

The seafood industry is often left out of the food waste discussion, but this sector is no exception, as it generates large amounts of various by-products. This study aimed to explore the potential of the microwave-assisted extraction (MAE) technique to obtain high-quality oil from fish by-products. The independent variables, which were time (1–30 min), microwave power (50–1000 W), and solid/liquid ratio (70–120 g/L) were combined in a 20-run experimental design coupled with the response surface methodology (RSM) for process optimization. The obtained oil yield values were fitted to a quadratic equation to build the theoretical models, which were statistically validated based on statistical criteria and used to predict the optimal MAE condition. The oil yields were significantly affected by the three independent variables through linear, quadratic, and/or interactive effects. Compared to a conventional Soxhlet extraction (SE), the optimal MAE conditions allowed between 60 and 100% of oil to be recovered in less than 19 min and with less solvent consumption. The fatty acid profiles of the oils obtained through SE and optimized MAE were characterized by gas chromatography with flame ionizing detection (GC-FID) after a derivatization process. These oils were constituted mainly of health, beneficial unsaturated fatty acids, such as oleic, docosahexaenoic (DHA), linoleic, and eicosapentaenoic (EPA) acids, which were not affected (*p* > 0.05) by the extraction methods. Interestingly, the oils obtained through MAE showed the best microbial growth inhibition results may have been due to thermolabile compounds, preserved via this unconventional non-thermal method. The oils also exhibited anti-inflammatory effects via nitric oxide production inhibition and cytotoxic potential especially, against breast and gastric adenocarcinoma cells. However, the threshold of toxicity should be further investigated. Overall, this work emerges as a future-oriented approach to upcycling fish by-products into high-quality oils that can be used in the formulation of pet food and other products.

## 1. Introduction

The agri-food industry generates many by-products with considerable economic, social, and environmental implications [1]. The upcycling of food by-products into value-added products has been a major challenge, both for the food industry and the scientific community, in order to achieve a circular economy through the efficient (re)use of natural resources [2]. This approach is relevant for the fish industry, since the production and processing of fish products leads to the disposal of more than 50% of the biomass annually. Solid fish waste consists of heads, tails, skin, viscera, fins, and bones, which can present a problem to the environment when discarded without any treatment [3]. In this context, several studies have been developed to explore the potential for application of these wastes in industry. The main nutritional constituents and bioactive properties of products resulting from the fish processing industry have been described [4].

Fishmeal and fish oil are currently the two main products resulting from the valorization of fish by-products [5]. Fish oils are an important source of long-chain polyunsaturated fatty acids (PUFAs) and include eicosapentaenoic acid (EPA; C20:5*n*3) and docosahexaenoic acid (DHA; C22:6*n*3), which are recognized as the most important *n*3 PUFAs due to their involvement in the prevention of cardiovascular diseases and other health conditions [6,7]. The fatty acid composition of fish oil varies depending on the species and other factors such as the fish’s environment and diet. In general, marine fish species have a higher content of *n*3 PUFAs due to their plankton-based diet, while freshwater fish have a higher monounsaturated fatty acid (MUFA) content due to the ingestion of surrounding vegetation [8]. The production of fish oil from fish processing waste appears to be a sustainable and future-oriented opportunity to provide valuable *n*3 PUFAs to both animal and human diets. Furthermore, the production of fish oil from fish side streams fulfills the goals of a circular economy, as waste or by-products from the industry become raw material for the production of other goods, thus entering the value chain and bringing several benefits [9].

At an industrial scale, rendering methods are the most commonly used conventional techniques for fish oil extraction [10]. However, these processes have some disadvantages and limitations that have driven the industry and the scientific community towards more profitable alternatives. Traditional oil extraction methods can degrade thermolabile PUFAs due to the high temperatures involved, during both wet and dry rendering processes. Furthermore, the chemicals used in some processes can result in residues in the final product, and may also cause environmental problems [11]. Enzyme extraction is a more recent technique that has been explored for this purpose, but which has been shown to be inefficient in generating satisfactory yields on an industrial scale [12]. Thus, it is important to develop clean and safe strategies to produce high-quality fish oil, increasing the efficiency of the extraction process and ensuring minimal environmental impact [13]. Different technologies have been explored to find the most efficient methodologies and conditions for extraction [14,15]. Recently, the unconventional microwave-assisted extraction (MAE) method has been investigated as a potentially sustainable method for extracting oil from fish by-products. According to de la Fuente et al. [16], MAE allows the recovery of more than 50% of the total lipid content from salmon backbones, heads, and viscera in less than 11 min of extraction. During MAE, microwaves promote an increase in pressure within the sample cells, leading to the rupture of their membranes and the consequent release of solutes into the solvent [17]. However, the potential of the MAE technique for extracting fish oil is still little explored. So far, MAE stands as an alternative technique mainly used for the recovery of chitosan from seafood waste [12].

This study was carried out to optimize the MAE of oil from category 3 fish by-products using an experimental design coupled with response surface methodology (RSM), a powerful statistical tool that describes the relationship between independent variables and one or more response variables, enabling process optimization with a reduced number of experimental trials. The oil yields obtained through the optimized MAE process were compared with those obtained through the conventional Soxhlet extraction (SE) method, and the nutritional quality of these oils was evaluated based on the fatty acid profile and lipid quality indices. In addition, antimicrobial effects against fungal and bacterial trains and in vitro antioxidant, anti-inflammatory, and cytotoxic properties were also evaluated and compared to assess the effect of the extraction method on these bioactivities.

## 2. Materials and Methods

### 2.1. Standards, Reagents, and Biological Material

The fatty acid methyl ester (FAME) reference standard mixture 37 (standard 47885-U), dexamethasone, quercetin, ellipticine, sulforhodamine B (SRB), trichloroacetic acid (TCA), tris(hydroxymethyl)aminomethane (Tris), dimethyl sulfoxide (DMSO), lipopolysaccharide (LPS), 2′,7′-dihydrodichlorofluorescein diacetate (DCFH-DA), and 2,2′-azobis(2-amidinopropane) dihydrochloride (AAPH) were provided by Sigma-Aldrich (St. Louis, MO, USA). Streptomycin and ampicillin were purchased from Fisher Scientific (Janseen Pharmaceutical, Beerse, Belgium). Ketoconazole and the bacteria and fungi used in the antimicrobial activity assays were obtained from Frilabo (Porto, Portugal). Muller-Hinton Broth (MHB) and Malt Extract Broth (MEB) were acquired from Biolab^®^ (Budapest, Hungary), while Blood Agar (Sheep blood 7%) was acquired from LiofilChemsrl (Roseto d. Abruzzi (TE), Italy). Sulfuric acid (98%), n-hexane (95%), methanol, toluene, and diethyl ether were provided by Fisher Scientific (Leicestershire, UK). The cell lines of gastric adenocarcinoma (AGS), colon adenocarcinoma (Caco-2), African green monkey kidney epithelium (VERO), and murine macrophages (RAW 264.7) were purchased from the European Collection of Authenticated Cell Cultures (ECACC, Salisbury, UK), while breast adenocarcinoma (MCF-7) and non-small cell lung cancer (NCI-H460) cells were purchased from the Leibniz-Institute DSMZ (Braunschweig, Germany). Water was treated using a Milli-Q water purification system (TGI Pure Water Systems, Greenville, SC, USA).

### 2.2. Fish By-Product and Sample Preparation

**Figure 1:**
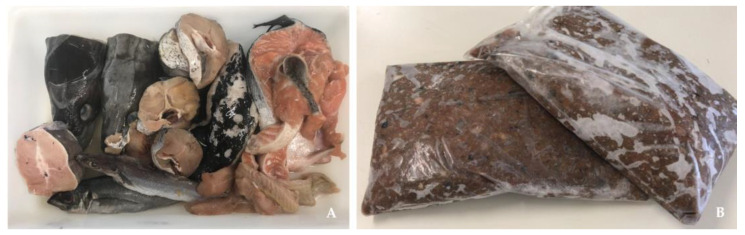
Industry-supplied fish by-product samples: unprocessed refrigerated sample, S1 (**A**); and frozen standard ground sample, S2 (**B**).

The fish by-products were provided by “Grupo ETSA—Empresa Transformadora de Subprodutos, S.A.” (Loures, Portugal), whose companies collect and transform various types of animal by-products into products for the animal feed and energy markets. The by-products supplied were category 3, i.e., quality by-products downgraded from food grade due to low commercial interest (such as the heads and fins of some species), breakage of the cold chain, or reaching the end of their shelf-life. Two batches of fish by-products were received, namely a refrigerated sample (S1) consisting of different species and parts of fish (36% salmon fillets, 28% European conger heads and loins, 12% whole blue whiting, 12% black swordfish fillets, 10% hake tails and fillets, 1% whole horse mackerel, and 1% perch loins) that were discarded due to reaching the end of their shelf life or a break in the cold chain (Figure 1A) and a frozen standard ground mixture (S2) representative of the by-products continuously collected by the company (Figure 1B). The use of these two samples was intended to verify whether the additional sample processing steps affected the lipid quality of the oils. Both samples were immediately lyophilized upon receipt at the lab and ground to a fine, homogeneous powder for the analyzes.

### 2.3. Experimental Design for MAE Optimization

A central composite rotatable design (CCRD) combining the independent variables *X*_1_ (time, *t*, 1–30 min), *X*_2_ (microwave power, *P*, 50–1000 W), and *X*_3_ (solid/liquid ratio, *R*, 70–120 g/L) was implemented to optimize the oil extraction from fish by-products, using RSM for process optimization. These independent variables and the respective range of values in Table 1 were selected based on data from the literature [16]. Design-Expert software, Version 11 (Stat-Ease, Inc., Minneapolis, MN, USA) was used to generate the 20-run CCRD, which included eight factorial points, six axial or star points, and one center point replicated six times. The runs were randomized to minimize the effects of unexpected variability.

### 2.4. Extraction Methods

#### 2.4.1. Microwave-Assisted Extraction (MAE)

The MAE process was conducted in a Nµ Tech microwave extractor (NuWav-Uno, Sonilex, West Bengal, India), whose technical specifications have been previously described [16]. The amount of fish by-product sample and *n*-hexane required to obtain the solid–liquid ratio defined in the experimental design, along with 50 mL of solvent, were combined in a flask and placed in the microwave chamber. The processing time and power were set by the digital panel. The microwave power was controlled by the Intelli-System based on real-time conditions to eliminate any temperature overshoot. Thus, 50 °C was the maximum temperature used in MAE. After processing, the mixtures were filtered and the solvent was separated using a rotary vacuum evaporator (Hei-VAP Silver 4, Schwabach, Germany), with the water bath at 40 °C. Extractions were performed at last in duplicate.

#### 2.4.2. Soxhlet Extraction (SE)

Conventional SE was used as a reference method for total oil extraction. The sample (5 g) and *n*-hexane (250 mL) were placed in the Soxhlet apparatus (Behr Labor Technik^TM^, Düsseldorf, Germany) at 20 g/L, and the oil was extracted, with the boiler at 80 °C for 6 h. The solvent was separated as described for MAE. Extractions were performed at least in triplicate.

### 2.5. Oil Yield Determination

For the two extraction methods, the resulting amount of oil extracted from the lyophilized (dw) fish by-products was calculated gravimetrically as follows:

(1) $$\mathrm{Yield}\;\left(g/100\;g\right)=\frac{\mathrm{weight}\;\mathrm{of}\;\mathrm{extracted}\;\mathrm{oil}}{\mathrm{weight}\;\mathrm{of}\;\mathrm{fish}\;\mathrm{material}}\times 100$$

### 2.6. MAE Optimization Using RSM

The MAE was optimized based on the fish oil yield (%, g/100 g dw) obtained over the 20 runs of the CCRD. For each fish by-product sample, the theoretical model was fitted by means of a least squares calculation using the second-order polynomial Equation (2):

(2) $$Y=b_{0}+b_{1}X_{1}+b_{2}X_{2}+b_{3}X_{3}+b_{11}X_{1}^{2}+b_{22}X_{2}^{2}+b_{33}X_{3}^{2}+b_{12}X_{1}X_{2}+b_{13}X_{1}X_{3}+b_{23}X_{2}X_{3}$$

where *Y* corresponds to the oil yield; *X*_1_, *X*_2_, and *X*_3_ define the independent variables time (*t*), microwave power (*P*), and solid/liquid ratio (*R*), respectively; *b_0_* is the constant coefficient; *b*_1_, *b*_2_, and *b*_3_ are coefficients of linear effect; *b*_11_, *b*_22_, and *b*_33_ are coefficients of quadratic effect; and *b*_12_, *b*_13_, and *b*_23_ are coefficients of interaction effect. For each term, the subscripts 1, 2, and 3 stand for time, microwave power, and solid/liquid ratio, respectively.

Fitting procedures, coefficient estimates, and statistical analysis were performed using Design-Expert software. Analysis of variance (ANOVA) was used to assess the significance of the models and their coefficients, as well as the lack-of-fit (which should have been non-significant (*p* > 0.05)). For the construction of the models, only significant terms (*p* < 0.05) and those required to ensure hierarchy were selected. The coefficient of determination (*R*^2^), adjusted coefficient of determination (*R*²_adj_), and adequate precision were used to estimate the adequacy of the polynomial equation to the response.

### 2.7. Experimental Validation of the Predictive Models

The optimal processing conditions that maximize fish by-product oil recovery using MAE were applied following the procedure described above. To validate the theoretical models, the experimentally obtained oil yield values were compared with the model-predicted ones through a post-analysis verification of Design-Expert (*p* < 0.05). These extractions were performed at least in triplicate to obtain enough oil for the analyses.

### 2.8. Evaluation of the Nutritional Properties of the Fish By-Product Oils

#### 2.8.1. Lipid Profile

Fatty acid methyl esters (FAMEs) were prepared from the fish by-product oils (500 µL) via transesterification with a methanol:sulfuric acid:toluene (2:1:1 *v*/*v*/*v*) catalytic solution (5 mL) overnight at 50 °C and 600 rpm. Then, 3 mL of water and 3 mL of diethyl ether were added and vortexed in order to achieve phase differentiation. FAMEs were recovered from the upper layer and mixed with sodium sulphate. After filtering through 0.22 µm nylon filters, the samples were diluted 1/10 in diethyl ether for injection. The fatty acid composition analysis was carried out using a gas chromatograph (GC) constituting of a DANI GC 1000 chromatograph (DANI Instruments, Milan, Italy), a flame ionization detector (FID), a split/splitless injector, and a Macherey-Nagel capillary column (30 m × 0.32 mm ID × 0.25 µm d_f_). Fatty acids were identified by comparing the relative retention times of FAME peaks from fish oils with the reference standard FAME mixture. Details of the analytical determinations have been described by Reis et al. [18].

#### 2.8.2. Lipid Quality Indices

The *n*3/*n*6 ratio, which is indicative of a healthy diet, was calculated from the relative percentages of fatty acids [19]. The atherogenicity (AI), thrombogenicity (TI), and hypocholesterolemic (HI) health indices of the fish by-product oils, linking fatty acid profile to cardiovascular risk [20,21], were calculated using the following equations:

(3) $$\mathrm{AI}=\frac{C12:0+\left(4\times C14:0\right)+C16:0}{\mathrm{MUFA}+\mathrm{PUFA}}$$

(4) $$\mathrm{TI}=\frac{C14:0+C16:0+C18:0}{\left(0.5\times \mathrm{MUFA}\right)+\left(0.5\times \mathrm{PUFA}\;n6\right)+\left(3\times \mathrm{PUFA}\;n3\right)+\frac{\mathrm{PUFA}\;n3}{\mathrm{PUFA}\;n6}}$$

(5) $$\mathrm{HI}=\frac{C18:1+\mathrm{PUFA}}{C12:0+C14:0+C16:0}$$

### 2.9. Evaluation of Bioactive Properties of Fish By-Product Oils

The bioactive properties of the fish by-product oils obtained through optimized MAE and SE were evaluated in vitro as described below.

#### 2.9.1. Antimicrobial Activity

The assays previously adapted by de la Fuente et al. [16] for salmon head oil were used to evaluate the antibacterial and antifungal activities of the oils. The Gram-negative bacteria *Enterobacter cloacae* (ATCC 49741), *Escherichia coli* (ATCC 25922), *Pseudomonas aeruginosa* (ATCC 9027), *Salmonella enterica* serotype Enteritidis (ATCC 13076), and *Yersinia enterocolitica* (ATCC 8610), and the Gram-positive bacteria *Bacillus cereus* (ATCC 11778), *Listeria monocytogenes* (ATCC 19111), and *Staphylococcus aureus* (ATCC 25923) were selected, as well as the micromycetes *Aspergillus fumigatus* (ATCC 204305) and *Aspergillus brasiliensis* (ATCC 16404). For antibacterial activity, 100 μL of oil was mixed with 100 μL of MHB with Tween 80 (0.1%) and then serially diluted in the same medium to obtain concentrations from 50% to 0.39%, to which 100 μL of inoculum at 1.5 × 10^5^ Colony Forming Unit (CFU)/mL was added. For antifungal activity, 100 μL of oil was mixed with 100 μL of MEB and serially diluted in the same medium. Culture media and oils were used as negative controls, while culture media and each inoculum, and the antibiotics ampicillin and streptomycin or the antifungal ketoconazole, were used as positive controls. The lowest concentrations that inhibited the visible microbial growth at the binocular microscope were assessed via color change and defined as minimum inhibitory concentrations (MIC). The minimum bactericidal and fungicidal concentrations (MBCs and MFCs) corresponded to the lowest oil concentration responsible for killing 99.5% of the inoculum after incubation of an aliquot from each well that showed no color change on solid medium.

#### 2.9.2. Cellular Antioxidant Activity (CAA)

The CAA assay previously adapted by de la Fuente et al. [16] for salmon head oil was used to evaluate the antioxidant activity of the fish oils at 500–2000 µg/mL in DMSO:H_2_O (1:1 *v*/*v*). This assay employs the cell-permeable fluorescent probe dye DCFH-DA, which can be diffused into RAW 264.7 cells and deacetylated by cellular esterases into the nonfluorescent polar derivative DCFH, which switches rapidly to the highly fluorescent DCF when oxidized by intracellular ROS and other peroxides. The fluorescence intensity was measured in a microplate reader (Biotek ELX800, Bio-Tek Instruments, Inc., Winooski, VT, USA) with fluorescence filters for an excitation wavelength of 485 nm and an emission wavelength of 535 nm at 37 °C. AAPH was used as a free radical generator, and quercetin at 0.3 µg/mL was used as a positive control. Results were expressed as the percentage of oxidation inhibition. 

#### 2.9.3. Anti-Inflammatory Activity

The procedure described by Sobral et al. [22] to measure the inhibition of nitric oxide (NO) produced by LPS-stimulated RAW 264.7 macrophages was followed to evaluate the anti-inflammatory potential of fish oils. The NO quantification was performed using a Griess Reagent System kit (Promega, Madison, WI, USA). Fish oils were dissolved in DMSO:H_2_O (1:1 *v*/*v*) at 8 mg/mL and successively diluted to the concentrations (6.25–400 µg/mL) to be tested. Dexamethasone at 50 µM was used as a positive control and oil samples without LPS were employed as negative controls. Results were expressed as the oil concentration (μg/mL) that caused 50% of NO production inhibition (IC_50_ value).

#### 2.9.4. Cytotoxic Activity

The SRB assay previously modified by de la Fuente et al. [16] for salmon by-product oils was applied to test the cytotoxic activity of the fish oils (at concentrations ranging from 6.25 to 400 µg/mL in DMSO:H_2_O (1:1 *v*/*v*)) on AGS, CaCo-2, MCF-7, and NCI-H460 tumor cell lines. Non-tumor PLP2 (the primary culture of porcine liver) and VERO cells were also used. Briefly, after cell trypsinization and seeding in microplates, 100 µL of cold 10% (*w*/*v*) TCA were added and then were incubated at 4 °C for 1 h. After removing the TCA, adhered cells were washed three times with water and dried. Cell staining was carried out by adding 100 µL of 0.057% (*w*/*v*) SRB solution at room temperature for 30 min. Excess dye was eliminated by washing with 1% (*v*/*v*) acetic acid. Next, 200 µL of 10 mM Tris base was used to dissolve the cells, and the absorbance of protein-bound dye was measured in a microplate reader at 510 nm. Ellipticine at 10 mM was used as a positive control. Plated cells without fish oil were used as a negative control, and their absorbance values were considered time zero for the calculations. Results were expressed as the extract concentration (μg/ mL) responsible for 50% of cell growth inhibition (GI_50_ value).

### 2.10. Statistical Analysis

Analytical determinations and bioassays were performed in triplicate, and results were expressed as mean ± standard deviation (SD) (except for antimicrobial activity). The SD values were rounded to one significant figure, which dictated the decimal place of the uncertain digit of the mean value. Statistical differences (*p* < 0.05) between two oil samples were assessed using Student’s t-test, while for three or more oil samples, a one-way analysis of variance was applied, and the dependent variables were compared using Tukey’s HSD test. Possible correlations between fatty acids and cytotoxic effects were assessed using Pearson’s correlation test. Tests were conducted using SPSS Statistics (IBM SPSS Statistics for Windows, Version 22.0. Armonk, North Castle, NY, USA: IBM Corp.).

## 3. Results and Discussion

### 3.1. MAE Process Optimization

The influence of the independent variables time, microwave power, and solid/liquid ratio on the MAE of oil from fish by-products was studied using RSM. The oil yield results obtained over the 20 runs of the experimental design from the two samples of fish by-products (S1 and S2) are shown in Table 2. The extraction yield varied with a function of the applied MAE conditions and ranged from 12.57 to 18.26 g/100 g dw in S1 and from 14.88 to 20.99 g/100 g dw in S2. Although the oil yields were higher from S2, the lowest and highest yields achieved with both fish by-product samples were obtained from the 5th and 13th runs of the experimental design, respectively. On the other hand, the GC-FID analysis showed that the fatty acid profile did not change significantly regardless of the applied MAE conditions (data not shown). Therefore, the extraction process was optimized when considering only the oil content as a dependent variable.

The experimental data in Table 2 were fitted to the second-order polynomial Equation (2) to construct the theoretical model demonstrated in Equations (6) and (7), which were used to predict the optimal MAE conditions that maximize oil recovery from the fish by-products S1 and S2, respectively.

(6) $$Y\;\left(S1\right)=14.36+0.78t+0.04P-1.50R+0.45R^{2}-0.3tR+0.37PR$$

(7) $$Y\;\left(S2\right)=19.5+0.52t+1.2P-1.0R-1.2t^{2}-1.0P^{2}-0.3PR$$

Equations (6) and (7) show that the three independent variables (*t*, *P*, and *R*) significantly affected (*p* < 0.05) the fish by-product oil extraction. The higher model coefficients translate to a more marked linear, quadratic, or interaction effect, regardless of its sign. Furthermore, the existence of interactions at least between two independent variables justified the use of RSM as an optimization tool, as one-factor-at-a-time approaches are unable to evaluate these terms and involve a greater number of tests, making them more costly and time-consuming.

**Figure 2:**
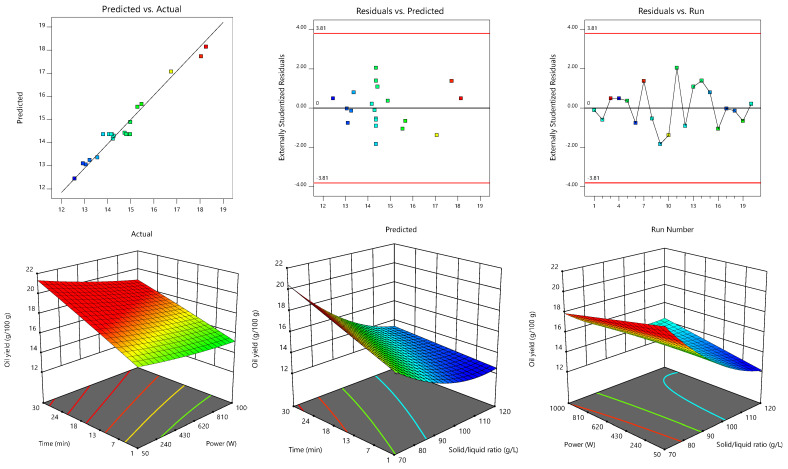
Response surface plots with statistical diagnostic representations illustrating the effects of the independent variables on oil yield (g/100 g) obtained from fish by-product S1 (unprocessed refrigerated sample). For each 3D plot, the excluded variable was fixed at its optimal value.

**Figure 3:**
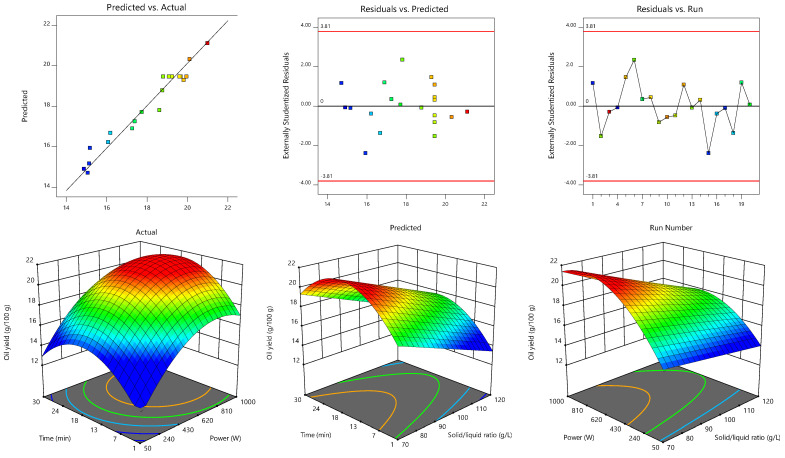
Response surface plots with statistical diagnostic representations illustrating the effects of the independent variables on oil yield (g/100 g) obtained from fish by-product S2 (frozen standard ground sample). For each 3D plot, the excluded variable was fixed at its optimal value.

The statistical data of the model fitting procedure are presented in Table 3, where one may observe that both models are statistically significant (*p* < 0.05) and present *R*^2^ and *R*²_adj_ values greater than 0.95 and 0.93, respectively, thus indicating that the variability is explained by the independent variables. *R*²_adj_ is lower than *R*^2^ because it penalizes the inclusion of terms that do not contribute to explaining the variability, and it is preferable whenever there are many terms in the model. This agreement between predicted and actual values is visually represented in the statistics diagnostic sections of Figure 2 and Figure 3, where the residual distribution is also shown to describe the behavior within the runs and show that there is no type of pattern that can influence the modeling. In addition, adequate precision values above 22.3 evidenced adequate model discrimination and the relatively low (≤2.7) coefficient of variation or relative standard deviation denoted a high degree of accuracy and reliability. A non-significant lack-of-fit (*p* > 0.05) also characterized both models, indicating that they fit the data in Table 2 well. Therefore, both models were statistically valid to navigate the design space and predict the optimal MAE conditions.

**Figure 4:**
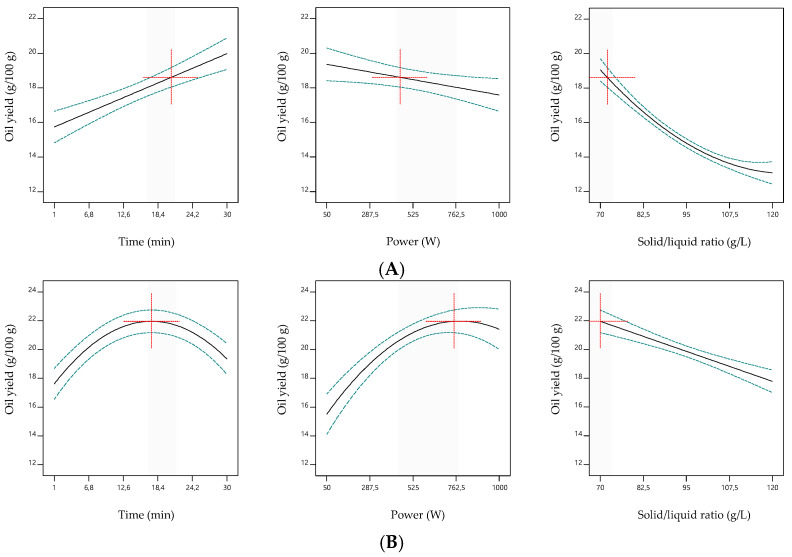
2D response graphs for the effects of the independent variables on the oil yield obtained from the fish by-products S1 (**A**) and S2 (**B**). For each graph, the excluded variables were fixed at their optimal value.

The 3D surface plots shown in Figure 2 and Figure 3 were constructed to visually represent the effects of the process variables on the oil content obtained from S1 and S2, respectively. For each 3D plot, the excluded variable was fixed at its optimal value. The extraction trends translated by the coefficients of Equation (6) and represented in Figure 2 for S1 showed the marked linear (−1.50) but also quadratic (0.45) effects of the solid/liquid ratio. Increases in this independent variable led to lower oil yields (since the *b*_3_ coefficient was negative). It also interacted with time (−0.3) and microwave power (0.37). Thus, the yield was promoted by the combination of low solid/liquid ratios with long irradiation times and low microwave powers. The extraction time also induced positive linear effects (0.78). Although the linear term coefficient (0.04) of microwave power was not significant (*p* > 0.05), it was necessary for the model hierarchy. In turn, Equation (7), which referred to the extraction of oil from the fish by-product S2, was more marked by quadratic terms (*b*_11_ = −1.2 and *b*_22_ = −1.0) than Equation (6), which are represented by the response surfaces in Figure 3 and by the individual 2D responses in Figure 4. The microwave power induced the strongest linear effects (1.2), followed by solid/liquid ratio (−1.0), and then time (0.52). An interaction effect (−0.3) between microwave power and solid/liquid ratio also characterized Equation (7).

In order to determine the optimal MAE conditions to maximize oil recovery from each fish by-product, the three independent variables were set within the experimental range and the response variable was “maximized.” The model Equations (6) and (7) estimated obtaining 18.6 ± 0.3 g/100 g dw and 22.0 ± 0.4 g/100 g dw of fish oil from S1 and S2, respectively, when processed at 455.5 W for 20.6 min at 72.1 g/L or at 750.2 W for 17.3 min at 70.0 g/L, respectively. The optimal conditions for time and solid/liquid ratio were similar for both samples, while the microwave power was the independent variable that most diverged. This deviation can be deduced from the 2D responses of each independent variable represented in Figure 2. As such, and since the batches of fish by-products generated by the industry can present certain compositional differences (e.g., variable proportions of different fish waste), global MAE conditions were determined considering the experimental data obtained with both samples, S1 and S2, simultaneously. This second optimization step showed that 18.3 ± 0.3 g/100 g dw and 21.4 ± 0.3 g/100 g dw of fish oil can be obtained from S1 and S2, respectively, when processed at 585.9 W for 18.7 min at a solid/liquid ratio of 70.9 g/L. Still, the oil yields obtained using these global conditions are similar to those predicted with the individual ones determined for each sample.

Some of the observed extraction trends can be compared with those described by de la Fuente et al. [16] for the MAE of oils from salmon side streams, namely higher oil yields when using lower solid/liquid ratios (~80 g/L for backbones and heads); medium processing times (~11 min for heads and ~14.5 min for backbones and viscera); and low or high microwave powers (50 W for heads and 961 W for viscera, though this variable was not significant for backbones). These results also show a greater discrepancy for the ultrasonic power, which corroborates our observations and may be justified by the variety and different intrinsic nature of the fish tissues that make up the by-products and their interaction with microwaves. In another work, Rahimi et al. [23] investigated the effects of MAE time on oil recovery from a homogenized mixture of fresh sardine heads, tails, and bones. Oil yields of 3.3 to 8.1 g/100 g fresh weight (fw) were obtained by increasing the irradiation time from 2 to 10 min using water as a solvent, while yields of 6.1 g/100 g fw were achieved by conducting irradiation over 4 min with a hexane:isopropanol ratio of 3:2 *v*/*v*. However, these were the maximum times tested by the authors, and extraction trends show that higher yields could be reached by processing longer. Still, the methods used by Soxhlet and by Hara and Radin yielded only 58% and 20% when compared to MAE.

### 3.2. Extraction Yields of Fish By-Product Oils Obtained through MAE and SE

To validate the predictive ability of Equations (6) and (7), the individual optimal MAE conditions discussed above were applied experimentally to obtain fish by-product oils. As shown in Table 4, 17.9 ± 0.8 g/100 g dw and 20.6 ± 0.9 g/100 g dw of fish by-product oil yields were obtained from S1 and S2, respectively, values which are in agreement with the model-predicted ones (18.6 ± 0.3 g/100 g dw and 22.0 ± 0.4 g/100 g dw), as confirmed by the post-analysis performed with the Design-Expert software. When comparing the S1 oil yields obtained through SE (18 ± 1 g/100 g dw) and optimized MAE, no statistically significant (*p* < 0.05) differences were found. However, for S2, the MAE process was only able to obtain 61% of the oil recovered from this sample using SE (34 ± 1 g/100 g dw). Even so, the shorter extraction times (17–21 times shorter) and lower solvent consumption (5 times less) associated with this non-conventional method stand out compared to SE.

The efficacy of MAE to extract fish oil was recently investigated using sea bream (*Sparus aurata*) and sea bass (*Dicentrarchus labrax*) heads [21]. The oil yield results (20.8–21.5 g/100 g dw), which corresponded to 52–55% of the total oil content, were similar to those of the present work. However, they were lower than those described by de la Fuente et al. [16] for salmon side streams (38–77 g/100 g dw). For instance, the best results were observed in salmon viscera, which reached 77 g/100 g (SE) and 71 g/100 g (optimized MAE), thus allowing 92% of the total oil to be recovered. For salmon backbones and heads, the oil recovered under optimized conditions was 69%. These MAE processes took up to 33 times less time and 5 times less solvent than SE. The potential impact of the matrix is thus somewhat observed. According to the microscopic observations made by Costa and Bragagnolo [24], MAE causes the rupture of fish tissue and consequent faster mass transfer, which explain the shorter extraction time required. In addition, MAE can reduce the extraction time by up to 90% and the solvent consumption by up to 25% compared to the conventional Folch method.

### 3.3. Fatty Acid Profile of the Fish By-Product Oils Obtained through MAE and SE

The fatty acid profile of the fish oil by-products obtained through SE and MAE consisted mainly of monounsaturated fatty acids (MUFAs, ~51%), given the high relative percentages of oleic acid (C18:1*n*9) (Table 4), an odorless olive oil associated with multiple beneficial effects on human health [25]. Palmitic (C16:0) and docosahexaenoic (DHA, C22:6*n*3) acids ranked second, with contents reaching ~14% (in S2 oil) and ~13% (in S1 oil), respectively. Relative percentages of linoleic (C18:2*n*6) and eicosapentaenoic (EPA, C18:2*n*6) acids up to 10 and 7%, respectively, and of α-linolenic acid (C18:3*n*3) up to 4.9% were also found in the studied fish oils. These last four fatty acids were the ones that most contributed to the 28–30% of polyunsaturated fatty acids (PUFAs). As shown in Table 4, neither the relative percentages nor the classes of fatty acids varied significantly (*p* > 0.05) with the extraction method. In general, the fatty acid composition of the studied oils was similar to that of oils from sea bass, sea bream, and salmon heads, as well as salmon backbones and viscera, all obtained through MAE and SE, in which oleic acid also stands as the most abundant (~34–39%), followed by linoleic (13–18%), palmitic (10–15%), docosahexaenoic (DHA, 7–14%), and eicosapentaenoic (EPA, 4–6%) acids [16,26]. These by-products thus had higher linoleic acid percentages than the by-products used in the present study. Oleic acid was also the main fatty acid in Atlantic salmon head, frame, and viscera oils (38.8–44.2%) obtained using enzymatic hydrolysis [11], and in cephalopod (*Sepioteuthis lessoniana*) liver viscera oil extracted using the Folch method [27]. According to Costa and Bragagnolo [24], the fatty acid composition of fish is not altered by the applied microwave energy. However, it is worth noting that MAE can be more suitable than extraction methods employing temperatures above 100 °C, as thermosensitive fatty acids such as EPA and DHA can be affected [28].

In addition to the high MUFA levels, the nutritional and functional value of the oils obtained from the fish by-products was also highlighted by the contents of DHA (11–13%) and EPA (~10%), two important α-linolenic acid derivatives found in marine fish oils and widely used because of their nutritional, medical, and healthcare value [29]. According to the Commission Regulation (EU) No 116/2010 on nutritional claims made on foods [30], the claim “high omega-3 fatty acids” can be attributed to foodstuffs containing at least 80 mg of EPA + DHA per 100 g and per 100 kcal. Therefore, both oils obtained from S1 and S2 complied with this claim since they contained about 6.0 and 4.1 g/100 g of EPA and DHA, respectively. The nutritional quality of these oils was also confirmed by the *n*6/*n*3 PUFA values, which were below 4 (namely from 0.62 to 0.73), as recommended by the Food and Agriculture Organization (FAO) [19]. Low atherogenicity (AI) and thrombogenicity (TI) indices and a high hypocholesterolemic index (HI) are considered beneficial for decreasing cardiovascular risk [31]. As shown in Table 4, the low atherogenic potential is indicated by AI values below 0.32, due to the greater amount of anti-atherogenic MUFAs and PUFAs compared to pro-atherogenic fatty acids (C14:0 and C16:0). The low thrombogenic potential (TI ≤ 0.26) resulted from the higher proportion of anti-thrombogenic fatty acids (MUFAs and *n*3 and *n*6 PUFAs) in relation to the pro-thrombogenic fatty acids identified in the oils. In turn, the HI values were equal to or higher than 3.7 (Table 4) and resulted from a relationship between hypocholesterolemic and hypercholesterolemic fatty acids. As verified for fatty acids, none of these lipid quality indices varied depending on the extraction method. In general, these indices are similar to those described by de la Fuente et al. [26] for oils from fish by-products.

### 3.4. Biological Activities of the Fish By-Product Oils Obtained through MAE and SE

#### 3.4.1. Antimicrobial Activity

The results of the antibacterial and antifungal activities of the fish by-product oils are shown in Table 5 as minimum inhibitory concentration (MIC) values. In general, the oils obtained through MAE presented better results than those obtained through SE. With few exceptions, 50 mg/mL of SE extracted oil were required to inhibit most of the tested bacteria. The Gram-negative bacillus-shaped *Yersinia enterocolitica* and the Gram-positive spherically shaped *Staphylococcus aureus* were the bacteria most sensitive to the MAE oils, with MIC values of 3.25 (for both S1 and S2) and 6.25 (for S1) and 3.125 (for S2), respectively. Both oils extracted through MAE yielded better results against *Enterobacter cloacae*, *Bacillus cereus*, *Salmonella enterocolitica*, and *Escherichia coli* than oils extracted through SE. Curiously, only the S2 oil obtained through MAE (at 6.25 mg/mL) inhibited *Listeria monocytogenes*, while 50 mg/mL of any oil was needed against *Pseudomonas aeruginosa* (being the most resistant bacterium among those tested). 

Since the fatty acid profile of the oils obtained through MAE and SE did not differ significantly (Table 4), the better effectiveness of the MAE recovered oils could be justified by the content of vitamins (such as A and E, among others [27]) or other thermolabile compounds that may have been preserved by this unconventional non-thermal method. The same conclusion was drawn in a study by la Fuente et al. [26], who also obtained low MIC values against *Y. enterocolitica* (3.125 mg/mL) and *S. aureus* (6.25/3.125 mg/mL) and a value of 50 mg/mL against *P. aeruginosa* with sea bass and sea bream head oils obtained through MAE. A good antibacterial activity of 1 mg/mL against *S. aureus*, *E. coli*, and *Proteus mirabilis* for cephalopod liver viscera oil was observed by Moovendhan et al. [27], who used the disk diffusion method. In a study by Som and Radhakrishnan [32], the maximum inhibitory effect of PUFAs recovered from saponified sardine oils was recorded against *P. aeruginosa*. However, the fish by-product oils of the present work were tested directly after extraction without converting the triglycerides into free fatty acids. There are also reports of the antimicrobial effects of commercially obtained EPA against the foodborne microorganisms *L. monocytogenes*, *S. aureus*, *S. aureus*, *P. aeruginosa*, and *Bacillus subtilis* [33].

Regarding antifungal activity, the fish by-product oils obtained through MAE also presented lower MIC values against *Aspergillus brasiliensis* (a common *Aspergillus* species associated with food contamination) and *A. fumigatus* (except for S2 oil) than oils obtained through SE. These results contrast with the findings reported by de la Fuente et al. [16], who attributed greater efficacy to salmon side stream oils extracted by SE than by MAE. Therefore, the panoply of bioactive compounds responsible for antifungal effects may be different from those responsible for the antibacterial activity. However, to the best of the authors’ knowledge, studies describing the antifungal activity of fish by-product oils are scarce.

In this work, minimum bactericidal/fungicidal concentrations (MIC and MFC) were also evaluated, but the maximum oil concentrations tested (50%) were not sufficient to kill any of the tested bacterial or fungal inoculum, which agrees with previous studies [16,26].

#### 3.4.2. Antioxidant and Anti-Inflammatory Activities

The antioxidant activity of the fish by-product oils was evaluated by a CAA assay. The oil obtained from S1 through MAE inhibited the oxidation reaction by 35%, while the other oils did not show antioxidant action at the maximum concentration tested (2000 µg/mL). With the same in vitro assay, de la Fuente et al. [16,26] observed up to 36% oxidation inhibition in salmon and sea bream head oils obtained through MAE, while those extracted by SE had no antioxidant effects. Additionally, while backbone oil showed no activity, viscera oil inhibited the cellular oxidation reaction by about 78%. Cephalopod liver oil extracted using the Folch method was also reported to display antioxidant effects via DPPH radical scavenging activity [27].

Table 6 shows the results of the anti-inflammatory activity of the fish by-product oils assessed via inhibition of NO production by LPS-stimulated murine macrophages. The oil concentrations needed to inhibit the NO production by 50% ranged from 11 to 20 µg/mL. The IC_50_ values varied depending on the extraction method, but also between samples in the case of MAE. These results are comparable to those previously described for sea bass and sea bream head oils (IC_50_ ranging from 14 to 21 µg/mL) [26], and better than that reported for oils from salmon heads (IC_50_ ranging from 51 to 75 µg/mL), backbones (IC_50_ ranging from 34 to 63 µg/mL), and viscera (IC_50_ ranging from 76 to 79 µg/mL), also obtained through SE and MAE [16]. In a study by Ahmad et al. [34], a correlation was observed between *n*3 PUFAs (such as EPA) in seafood lipid extracts and NO inhibition in LPS-stimulated RAW 264.7 cells, thus indicating that fatty acid composition can influence the anti-inflammatory activity.

#### 3.4.3. Cytotoxic Activity

The cytotoxic potentials of the obtained fish by-product oils were tested against tumor and nontumor cell lines and the results are shown in Table 6. All tested oils showed cytotoxic effects against all studied tumor cell lines. For breast adenocarcinoma (MCF-7), oils from S2 gave lower GI_50_ values (56–67 µg/mL) than those from S1 (218–233 µg/mL). According to a Pearson analysis, this result can be strongly correlated to the contents of stearic acids (C18:0, *r* = −0.858, *p* < 0.001), arachidonic acid (C20:4*n*6, *r* = −0.811, *p* = 0.001), dihomo-γ-linolenic acid (C20:3*n*6, *r* = −0.762, *p* < 0.004), and EPA (*r* = −0.703, *p* < 0.011). The growth of gastric adenocarcinoma cells (AGS) was also inhibited and found to moderately correlate (*r* = −0.672, *p* = 0.017) with EPA. For this cell line, which is extensively used in preclinical studies of stomach cancer, the highest GI_50_ value (274 µg/mL) was obtained for S1 oil obtained through SE, which also caused a slightly higher GI_50_ value (252 µg/mL) in non-small cell lung cancer (NCI-H460). In turn, colon adenocarcinoma (Caco-2) was the cell line least susceptible to the tested oils, as was also observed by de la Fuente et al. [16] for salmon side stream oils, and no statistical differences were observed between the oils. 

As shown in Table 6, the fish by-product oils also display cytotoxicity on non-tumor PLP2 (porcine liver) and VERO (monkey kidney epithelium) cells. Thus, the cytotoxicity of the oils tested was not selective for tumor cells, which is in agreement with previous studies carried out with oils from fish by-products [16,26]. Furthermore, VERO cells tended to be more sensitive to the oils than PLP2 through effects that could be strongly correlated (*r* = −0.959, *p* = 0.002) with heptadecanoic acid (C17:0), also known as margaric acid. The cytotoxicity of this odd-chain fatty acid has also been observed in normal mammary epithelial cells (MCF-10A) [35].

The possible preventive and therapeutic role of *n*3 PUFAs against various types of cancer has been demonstrated by different in vitro studies, which described complex molecular pathways modulated by these fatty acids [36]. Therefore, the mechanisms involved in the cytotoxicity of fish by-product oils must be studied in detail to understand the toxicity threshold for tumor and non-tumor cells.

## 4. Conclusions

Compared to traditional SE, the MAE technique allowed for the recovery of 60–100% of the total oil content from fish by-products in less than 19 min and with lower solvent consumption. The obtained oils consisted of approximately 51% MUFAs (mostly oleic acid), 29% PUFAs (including DHA, linoleic acid, and EPA), and 20% SFA (mostly palmitic acid), a profile that highlighted their potential protective effect with regard to cardiovascular risk. Although the fatty acid profile of the oils was not significantly affected by the extraction method, those obtained through MAE showed the best microbial growth inhibition results, which could be attributed to thermolabile constituents possibly degraded by SE. The oils also exhibited anti-inflammatory effects via NO production inhibition and cytotoxic potential, especially against breast and gastric adenocarcinoma cells. However, the threshold of toxicity should be further investigated, as well as other oil constituents and their oxidative stability. Overall, this work represents a future-oriented approach towards fish by-product valorization through a circular economy approach.

## Figures and Tables

**Table 1:** Natural and coded values of the independent variables used in the CCRD to optimize the MAE of oil from fish by-products.

Coded Values	Natural Values		
	*X*_1_: *t* (min)	*X*_2_: *P* (W)	*X*_3_: *R* (g/L)
−1.68	1	50	70
−1	7	243	80
0	15.5	525	95
+1	24	807	110
+1.68	30	1000	120

*t*: time; *P*: microwave power; *R*: solid/liquid ratio.

**Table 2:** Oil content obtained under the MAE conditions defined by the experimental design.

Run	Experimental Domain			Experimental Response (Oil Yield)	
	*X*_1_: *t* (min)	*X*_2_: *P* (W)	*X*_3_: *R* (g/L)	S1 (g/100 g dw)	S2 (g/100 g dw)
1	7	243	80	15.29	16.08
2	24	243	80	18.04	17.39
3	7	807	80	14.98	19.82
4	24	807	80	16.75	20.11
5	7	243	110	12.57	14.88
6	24	243	110	13.56	15.17
7	7	807	110	13.22	16.19
8	24	807	110	14.23	17.74
9	1	525	95	13.05	15.14
10	30	525	95	15.47	17.27
11	15.5	50	95	14.26	15.07
12	15.5	1000	95	14.74	18.76
13	15.5	525	70	18.26	20.99
14	15.5	525	120	12.93	18.62
15	15.5	525	95	14.06	19.95
16	15.5	525	95	14.18	19.67
17	15.5	525	95	14.16	18.80
18	15.5	525	95	14.81	19.61
19	15.5	525	95	13.80	19.08
20	15.5	525	95	14.97	19.24

*t*: time; *P*: microwave power; *R*: solid/liquid ratio.

**Table 3:** Statistical information of the model fitting procedure.

Statistical Criteria	S1 (Equation (6))	S2 (Equation (7))
Model F-value	60.79	49.35
Lack of Fit	0.9307	0.3477
*R* ^2^	0.9656	0.9579
*R* ^2^_adj_	0.9497	0.9385
Adequate Precision	27.83	22.32
Coefficient of Variation	2.36	2.70

*R*²: coefficient of determination; *R*²_ajd_: adjusted coefficient of determination.

**Table 4:** Yield and fatty acid profile of the oils extracted from fish by-products using Soxhlet extraction (SE) and optimized microwave-assisted extraction (MAE).

		SE		MAE	
		S1 Oil	S2 Oil	S1 Oil	S2 Oil
Oil yield (g/100 g dw)		18 ± 1 ^b^	34 ± 1 ^a^	17.9 ± 0.8 ^b^	20.6 ± 0.9 ^b^
Fatty acids (%)					
Myristic acid	C14:0	2.80 ± 0.07 ^a^	2.77 ± 0.05 ^a^	2.77 ± 0.07 ^a^	2.75 ± 0.06 ^a^
Pentadecanoic acid	C15:0	0.272 ± 0.06 ^a^	0.279 ± 0.007 ^a^	0.267 ± 0.007 ^a^	0.278 ± 0.006 ^a^
Palmitic acid	C16:0	12.3 ± 0.3 ^b^	14.2 ± 0.4 ^a^	12.2 ± 0.4 ^b^	14.1 ± 0.3 ^a^
Palmitoleic acid	C16:1	3.8 ± 0.1 ^a^	3.8 ± 0.1 ^a^	3.66 ± 0.04 ^a^	3.7 ± 0.1 ^a^
Heptadecanoic acid	C17:0	0.187 ± 0.004	nd	0.181 ± 0.005	nd
Heptadecenoic acid	C17:1	0.218 ± 0.006	nd	0.192 ± 0.005	nd
Stearic acid	C18:0	2.97 ± 0.04 ^b^	3.87 ± 0.09 ^a^	2.99 ± 0.06 ^b^	3.96 ± 0.08 ^a^
Oleic acid	C18:1*n*9	36.0 ± 0.9 ^a^	34.9 ± 0.8 ^a^	36 ± 1 ^a^	34.8 ± 0.8 ^a^
Linoleic acid	C18:2*n*6	9.9 ± 0.1 ^a^	10.1 ± 0.3 ^a^	10.3 ± 0.3 ^a^	10.1 ± 0.3 ^a^
γ-Linoleic acid	C18:3*n*6	0.15 ± 0.01 ^c^	0.193 ± 0.005 ^a^	0.103 ± 0.002 ^d^	0.170 ± 0.003 ^b^
α-Linolenic acid	C18:3*n*3	4.9 ± 0.1 ^a^	4.35 ± 0.05 ^b^	4.76 ± 0.07 ^a^	4.3 ± 0.1 ^b^
Eicosenoic acid	C20:1	3.12 ± 0.06 ^a^	3.04 ± 0.08 ^a^	3.06 ± 0.07 ^a^	3.03 ± 0.09 ^a^
Eicosadienoic acid	C20:2*n*6	0.95 ± 0.02 ^a^	0.77 ± 0.02 ^c^	0.86 ± 0.02 ^b^	0.76 ± 0.02 ^c^
Dihomo-γ-linolenic acid	C20:3*n*6	0.194 ± 0.006 ^b^	0.225 ± 0.006 ^a^	0.193 ± 0.005 ^b^	0.23 ± 0.01 ^a^
Arachidonic acid	C20:4*n*6	0.659 ± 0.008 ^b^	0.87 ± 0.02 ^a^	0.680 ± 0.007 ^b^	0.90 ± 0.02 ^a^
Eicosatrienoic acid	C20:3*n*3	0.50 ± 0.01 ^b^	0.408 ± 0.008 ^c^	0.53 ± 0.02 ^a^	0.39 ± 0.01 ^c^
Eicosapentaenoic acid	C20:5*n*3	6.3 ± 0.2 ^b^	7.0 ± 0.2 ^a^	6.7 ± 0.2 ^a,b^	7.0 ± 0.2 ^a^
Nervonic acid	C24:1	1.86 ± 0.05 ^a^	1.89 ± 0.05 ^a^	1.9 ± 0.1 ^a^	1.94 ± 0.07 ^a^
Docosahexaenoic acid	C22:6*n*3	12.9 ± 0.3 ^a^	11.3 ± 0.3 ^b^	12.8 ± 0.4 ^a^	11.5 ± 0.3 ^b^
Fatty acid class					
Saturated fatty acids	SFA	18.5 ± 0.4 ^b^	21.1 ± 0.3 ^a^	18.4 ± 0.5 ^b^	21.1 ± 0.4 ^a^
Monounsaturated fatty acids	MUFA	51 ± 1 ^a^	50.6 ± 0.9 ^a^	51.4 ± 0.9 ^a^	50.5 ± 0.4 ^a^
Polyunsaturated fatty acids	PUFA	30.1 ± 0.5 ^a^	28.3 ± 0.7 ^b^	30.22 ± 0.02 ^a^	28.4 ± 0.2 ^b^
	*n*3	18.3 ± 0.6 ^a^	16.1 ± 0.5 ^b^	18.1 ± 0.5 ^a^	16.2 ± 0.3 ^b^
	*n*6	11.4 ± 0.6 ^a^	11.8 ± 0.1 ^a^	12 ± 1 ^a^	11.8 ± 0.1 ^a^
	*n*9	36 ± 1 ^a^	35 ± 1 ^a^	36 ± 1 ^a^	35 ± 1 ^a^
Lipid quality indices					
*n*6/*n*3 PUFA		0.62 ± 0.05 ^b^	0.73 ± 0.03 ^a^	0.65 ± 0.08 ^a,b^	0.73 ± 0.01 ^a^
Atherogenicity index	AI	0.29 ± 0.01 ^b^	0.32 ± 0.01 ^a^	0.29 ± 0.002 ^b^	0.32 ± 0.01 ^a^
Thrombogenicity index	TI	0.21 ± 0.01 ^b^	0.26 ± 0.01 ^a^	0.21 ± 0.01 ^b^	0.26 ± 0.01 ^a^
Hypocholesterolemic index	HI	4.4 ± 0.1 ^a^	3.7 ± 0.1 ^b^	4.4 ± 0.3 ^a^	3.7 ± 0.1 ^b^

In each line, different letters indicate significant differences (*p* < 0.05) among oil samples. For C17:0 and C17:1, no significant differences (*p* > 0.05) were detected. nd: not detected.

**Table 5:** Antimicrobial activity of the oils extracted from fish by-products using Soxhlet extraction (SE) and optimized microwave-assisted extraction (MAE). The results are presented as minimum inhibitory concentration (MIC, mg/mL) values.

	SE		MAE		Positive Controls	
	S1 Oil	S2 Oil	S1 Oil	S2 Oil	Streptomycin	Ampicillin
Gram-negative bacteria						
*Enterobacter cloacae*	50	50	12.5	3.125	0.007	0.15
*Escherichia coli*	50	50	25	12.5	0.01	0.15
*Pseudomonas aeruginosa*	>50	50	50	50	0.06	0.63
*Salmonella enterocolitica*	50	25	25	6.25	0.007	0.15
*Yersinia enterocolitica*	50	50	3.25	3.25	0.007	0.15
Gram-positive bacteria						
*Bacillus cereus*	50	50	12.5	12.5	0.007	nt
*Listeria monocytogenes*	50	50	50	6.25	0.007	0.15
*Staphylococcus aureus*	25	50	6.25	3.125	0.007	0.15
					Ketoconazole	
*Aspergillus brasiliensis*	10	>10	10	5	0.06	-
*Aspergillus fumigatus*	>10	10	5	>10	0.5	-

The oil samples were tested at concentrations ranging from 50% to 0.39% (*v*/*v*). nt: not tested.

**Table 6:** NO production inhibition capacity (IC_50_ values, μg/mL) and cytotoxic activity to tumor and non-tumor cell lines (GI_50_ values, µg/mL) of the oils extracted from fish by-products through Soxhlet extraction (SE) and optimized microwave-assisted extraction (MAE).

	SE		MAE		Positive Control *
	S1 Oil	S2 Oil	S1 Oil	S2 Oil	
NO production inhibition	14 ± 1 ^c^	12 ± 1 ^b,c^	11 ± 1 ^b^	20.0 ± 0.4 ^d^	6.3 ± 0.4 ^a^
Tumor cell lines					
AGS	274 ± 9 ^d^	137 ± 8 ^b^	173 ± 2 ^b,c^	201 ± 15 ^c^	1.23 ± 0.03 ^a^
CaCo-2	316 ± 26 ^b^	302 ± 27 ^b^	302 ± 4 ^b^	316 ± 26 ^b^	1.21 ± 0.02 ^a^
MCF-7	233 ± 22 ^c^	56 ± 4 ^b,c^	218 ± 5 ^c^	67 ± 1 ^a,b^	1.02 ± 0.02 ^a^
NCI-H460	252 ± 12 ^c^	241 ± 17 ^b,c^	209 ± 14 ^b^	226 ± 16 ^b,c^	1.01 ± 0.01 ^a^
Nontumor cell lines					
PLP2	267 ± 21 ^c^	118 ± 7 ^b^	190 ± 12 ^b^	201 ± 11 ^b,c^	1.41 ± 0.06 ^a^
VERO	150 ± 13 ^b^	125 ± 6 ^b^	164 ± 13 ^b^	180 ± 8 ^b^	1.41 ± 0.06 ^a^

* Dexamethasone for the NO production inhibition assay and ellipticine for the cytotoxic activity assay. In each line, different letters translate significant differences (*p* < 0.05) among oil samples.

## Data Availability

Not applicable.
